# Lp16-PSP, a Member of YjgF/YER057c/UK114 Protein Family Induces Apoptosis and p21^WAF1/CIP1^ Mediated G_1_ Cell Cycle Arrest in Human Acute Promyelocytic Leukemia (APL) HL-60 Cells

**DOI:** 10.3390/ijms18112407

**Published:** 2017-11-13

**Authors:** Thomson Patrick Joseph, Warren Chanda, Abdullah Faqeer Mohammad, Sadia Kanwal, Samana Batool, Meishan Zhang, Mintao Zhong, Min Huang

**Affiliations:** 1 Department of Microbiology, College of Basic Medical Sciences, Dalian Medical University, Dalian 116044, Liaoning, China; microbiologist02@gmail.com (T.P.J.); chandawarren@yahoo.com (W.C.); samana.batool12@yahoo.com (S.B.); 18940876833@163.com (M.Z.); dyzhongmt@163.com (M.Z.); 2 Institute of Cancer Stem Cell, Dalian Medical University, Dalian 116044, Liaoning, China; abdullah.faqeer.mohammad@gmail.com; 3 Department of Biotechnology, College of Basic Medical Sciences, Dalian Medical University, Dalian 116044, Liaoning, China; sadiakanwal22@yahoo.com

**Keywords:** *Lentinula edodes*, Lp16-PSP, acute promyeloid leukemia, extrinsic and intrinsic apoptotic pathway, G_1_ phase cell cycle arrest, NF-κB signaling pathway

## Abstract

Lp16-PSP (Latcripin 16-Perchloric acid Soluble Protein) from *Lentinula edodes* strain C_91-3_ has been reported previously in our laboratory to have selective cytotoxic activity against a panel of human cell lines. Herein, we have used several parameters in order to characterize the Lp16-PSP-induced cell death using human acute promyeloid leukemia (HL-60) as a model cancer. The results of phase contrast microscopy, nuclear examination, DNA fragmentation detection and flow cytometry revealed that high doses of Lp16-PSP resulted in the induction of apoptosis in HL-60 cells. The colorimetric assay showed the activation of caspase-8, -9, and -3 cascade highlighting the involvement of Fas/FasL-related pathway. Whereas, Western blot revealed the cleavage of caspase-3, increased expression of Bax, the release of cytochrome c and decreased expression of Bcl-2 in a dose-dependent manner, suggesting the intrinsic pathway might be involved in Lp16-PSP-induced apoptosis as well. Low doses of Lp16-PSP resulted in the anchorage-independent growth inhibition, induction of G_1_ phase arrest, accompanied by the increased expression of p21^WAF1/CIP1^, along with the decreased expression of cyclin D, E, and cdk6. In addition, Lp16-PSP resulted in constitutive translocation inhibition of transcription factor nuclear factor kappa B (NF-κB) into the nucleus by decreasing the phosphorylation of IκBα. All these findings suggested Lp16-PSP as a potential agent against acute promyeloid leukemia; however, further investigations are ultimately needed.

## 1. Introduction

In the United States, approximately every three minutes a person is diagnosed with hematological cancer [1]. Leukemia is one type of blood cancer that usually initiates in blood-forming organs, including bone marrow, followed by the increment in abnormal leukocyte numbers. On the basis of pathological features, leukemia can be classified as acute and chronic leukemia, where acute leukemia can be acute myeloid leukemia (AML) or acute lymphoblastic leukemia (ALL) and, on the other hand, chronic leukemia can be chronic myeloid leukemia (CML) or chronic lymphocytic leukemia (CLL) [2]. As far as acute myeloid leukemia (AML) is concerned it is characterized by malignant hematopoietic progenitor cell (HPC) accumulation that has impaired the differentiation program [3]. In the United States alone, around 21,380 new cases of acute myeloid leukemia (AML) are expected to occur in the year 2017 with an overall five-year survival rate of 26.9% [1].

Acute promyeloid leukemia (APL) is the clear subtype of AML, which is caused by leukocyte differentiation arrest at the promyelocyte stage and was considered as fatal before the discovery of all-trans retinoic acid (ATRA), the derivative of vitamin A [4]. In the treatment of APL patients, risk stratification is considered to be crucial, and so less intensive regimens are adapted for treating the patients at low risk, having white blood cell counts (WBC) of ≤10,000/µL, in comparison with patients presenting high-risk disease (WBC ≈ 410,000/μL). Initially, APL patients were defined as low-risk for relapse (WBC ≤ 10,000/μL and platelet count ≈ 440,000/μL), intermediate-risk (WBC ≤ 10,000/μL and platelet count ≤ 40,000), and high-risk (WBC ≈ 410,000/μL) on the basis of cell count [5]. However, because low- and intermediate-risk patients have common outcomes, they were collectively considered as a low-risk disease. As far as therapy for newly-diagnosed APL patients is concerned, in the last two decades, it has excogitated from an all-trans retinoic acid (ATRA) + chemotherapy to the arsenic trioxide (ATO) addition, followed by the chemotherapy omission in low-risk patients [6].

Natural products, from higher plants, fungi, and microorganisms, have a long history of being used as therapeutic agents for the treatment of several diseases. The compounds from natural resources have proven their therapeutic role in unmodified form, as well as in contributing a variety of derivatives called secondary metabolites [7]. Concerning the role of natural products in the treatment of leukemia, l-asparaginase, daunorubicin, and the anthracyclines are well-known for their anti-leukemia activities [8]. Success of the treatment not just depends on the category, but also on the genetic factors associated with each disease. Chemotherapy, radiation therapy, antibiotics usage, transfusion, and transplantation of blood and bone marrow are some of the strategies used in combination to treat leukemia patients, respectively. Although these strategies resulted in prolonged survival, however, some of these treatments are difficult to handle [9]. In order to make advances in our journey toward curative therapy, there is a continuous need of identifying the novel protective mechanisms and pathways responsible for the survival of tumor, as we cannot only rely on the presently available arsenals.

In general, if we look into the strategies for combating cancer, in the flow of genetic information, the expression of the gene(s) in the cancer cells can be controlled at different levels, but the drug that targets DNA have the disadvantage of being mutagenic. In contrast, the agents that target RNA are significantly effective without having genotoxic effects [10]. Current strategies to combat cancer are not always effective, because of the development of resistance, severe side effects, and diversity of the targets in the cancer cells. Cancer biologists are targeting the RNA pool of cancer cells as alternative strategies for combating cancer, as it differs significantly from the normal cells highlighting the additive advantage of selective toxicity. In this regard, many of the characterized ribonucleases from various organisms have displayed antitumor activities [11,12,13,14] and efforts to increase such arsenals are still ongoing, especially with natural resources.

Keeping in mind this alternative strategy to combat cancer, and based on the bioinformatics analysis, our laboratory has demonstrated that Lp16-PSP from the edible mushroom *Lentinula edodes* C_91-3_ is an endoribonuclease L-PSP and is a member of the highly-conserved YjgF/YER057c/UK114 protein family [15]. The members of YjgF/YER057c/UK114 family are the small protein found in three domains of life with 4–6 conserved amino acid residues and reported to be involved in several biological processes [16,17,18]. A rat liver perchloric acid-soluble protein was characterized as an endoribonuclease and was reported to inhibit the initiation stage of protein translation in rabbit reticulocyte lysate systems [16,19]. Later in this series, hp14.5, a homologue of rat liver perchloric acid-soluble protein, UK114, and a bovine homologue were reported as a translation inhibitor [20], antineoplastic and tumor antigen [21,22,23,24], and activator of calpains, respectively [25]. In addition to the mammalian protein members, other proteins identified in bacteria and eukaryotes include the purine regulator YabJ from *Bacillus subtilus* [26], YIL051c and YER057c from *Saccharomyces cerevisiae* involved in the mitochondrial biosynthesis and maintenance [27], and the plant’s protein that has a role in photosynthesis/chromoplastogenesis [28]. In the recent past, the antiviral activity of the endoribonuclease L-PSP protein has been reported that was isolated from the bacterium *Rhodopseudomonas palustris* strain JSC-3b [29]. Moreover, repression of cell proliferation and fatty acid binding ability of the members of YjgF/ YER057c/UK114 superfamily has also been reported [30,31]. In our previous study, we have demonstrated the selective anticancer activity of Lp16-PSP against a panel of human cell lines and acute promyeloid leukemia HL-60 cell line was identified as the most sensitive cell line with the IC_50_ value of 74.4 ± 1.07 µg/mL after 48 h of treatment [15]. Therefore, the objective of this study is to use human acute promyeloid leukemia (HL-60 cells) as a model cancer to further investigate the potential molecular mechanism of the action of Lp16-PSP. We, thus, investigated several parameters, such as DNA fragmentation, mitochondrial membrane potential, *Bax*/*Bcl-2* expression, activation of caspases, and cell cycle distribution, in HL-60 cells as an in vitro model system. In this study, we observed that Lp16-PSP resulted in the increased expression of *FasL*, together with the loss of mitochondrial membrane potential and the release of cytochrome c, indicating that extrinsic and intrinsic pathways might be involved in the induction of apoptosis. In addition, Lp16-PSP also resulted in the anchorage-independent growth inhibition and p21^WAF1/CIP1^-mediated G_1_ cell cycle arrest in HL-60 cells. Furthermore, our findings demonstrated that the effect and molecular mechanism behind the action of Lp16-PSP are associated with the inhibition of the constitutive translocation of NF-κB into the nucleus by decreasing the phosphorylation of IκBα in human acute premyeloid leukemia (HL-60 cells).

## 2. Results

### 2.1. Cytotoxic Activity of Lp16-PSP in Human Acute Promyeloid Leukemia (HL-60 Cells)

**Figure 1:**
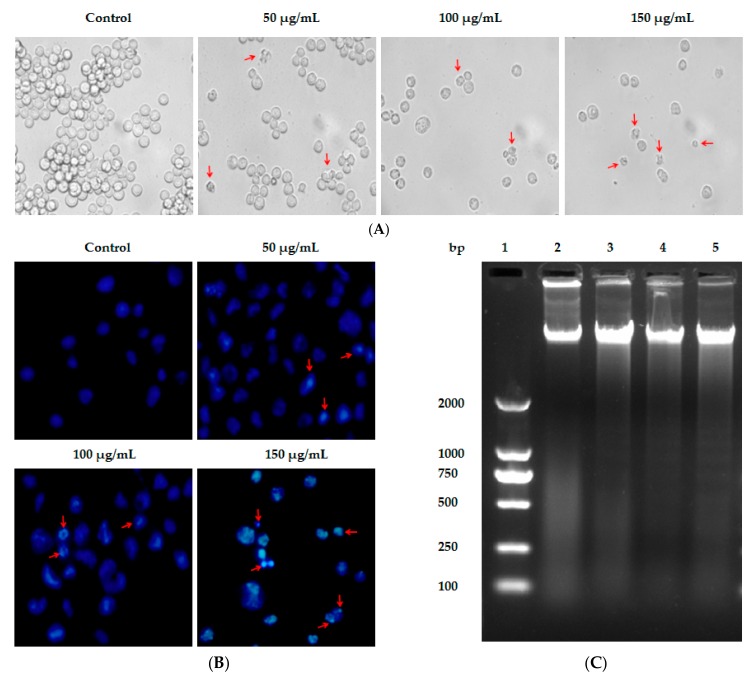
Lp16-PSP-induced cytotoxicity, nuclear morphological change and DNA fragmentation in acute promyeloid leukemia (HL-60 cells). (**A**) Phase contrast image of HL-60 cells, untreated and treated samples after 48 h of Lp16-PSP treatment. Red arrowheads are indicating the cells smaller in size and also showing cellular bleeding at 40× magnification; (**B**) fluorescent images of Hoechst 33258 stained HL-60 cells for nuclear morphological changes after 48 h of Lp16-PSP treatment with indicated concentrations at 40× magnification; and (**C**) DNA fragmentation of HL-60 cells treated with 0 µg/mL, 50 µg/mL, 100 µg/mL, and 150 µg/mL of Lp16-PSP for 48 h. Lane 1: DNA marker DL2000; lane 2: 0 µg/mL; lane 3: 50 µg/mL; lane 4: 100 µg/mL; and lane 5: 150 µg/mL Lp16-PSP exposed group. Results are representative of three independent experiments.

After treatment of the HL-60 cells with indicated concentrations of Lp16-PSP, phase contrast images were taken. As shown in Figure 1A, treated HL-60, in comparison with the untreated group, shows an obvious change in morphology, cell volume, and size. The cells in the treated group are smaller in size and also show the cellular bleeding. All these signs indicate that the HL-60 cells are going through the process of apoptosis or are dead. These findings highlighted the implication of Lp16-PSP as a potential anticancer agent against acute promyeloid leukemia (APL).

### 2.2. Lp16-PSP-Induced Nuclear Morphological Change and DNA Fragmentation in Acute Promyeloid Leukemia (APL) HL-60 Cells

Apoptosis or programmed cell death is characterized by certain typical features, i.e., shrinkage of the cell, condensation of nuclear chromatin, cleavage of chromosomes, bleeding of the membrane, and formation of apoptotic bodies [32,33].

In this study, Hoechst 33258 assay was used to monitor changes in the nucleus of HL-60 cells induced after treated with Lp16-PSP. Hoechst 33258 is a DNA-specific fluorochrome which upon excitation with UV emits a blue fluorescence. As shown in Figure 1B, the nuclei of untreated HL-60 cells are round with homogenous blue fluorescence, whereas Lp16-PSP-exposed cells showed shrinkage, nuclear chromatin condensation, and apoptotic body formation. The oligonucleosomal fragmentation of chromosomal DNA is another biochemical feature of apoptosis [34,35,36] that was studied by using DNA fragmentation assay after Lp16-PSP treatment of HL-60 cells for 48 h. DNA was extracted from HL-60 cells and studied using agarose gel electrophoresis. The electrophoretogram given shows the fragmentation of DNA, while no significant “DNA ladder-like” pattern was found in the control group (Figure 1C). Moreover, a concentration-dependent increase in DNA cleavage was also observed for the Lp16-PSP-treated samples.

### 2.3. Lp16-PSP-Induced Apoptosis and Involvement of Extrinsic and Intrinsic Pathways

**Figure 2:**
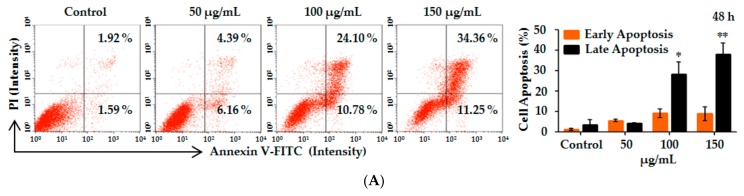

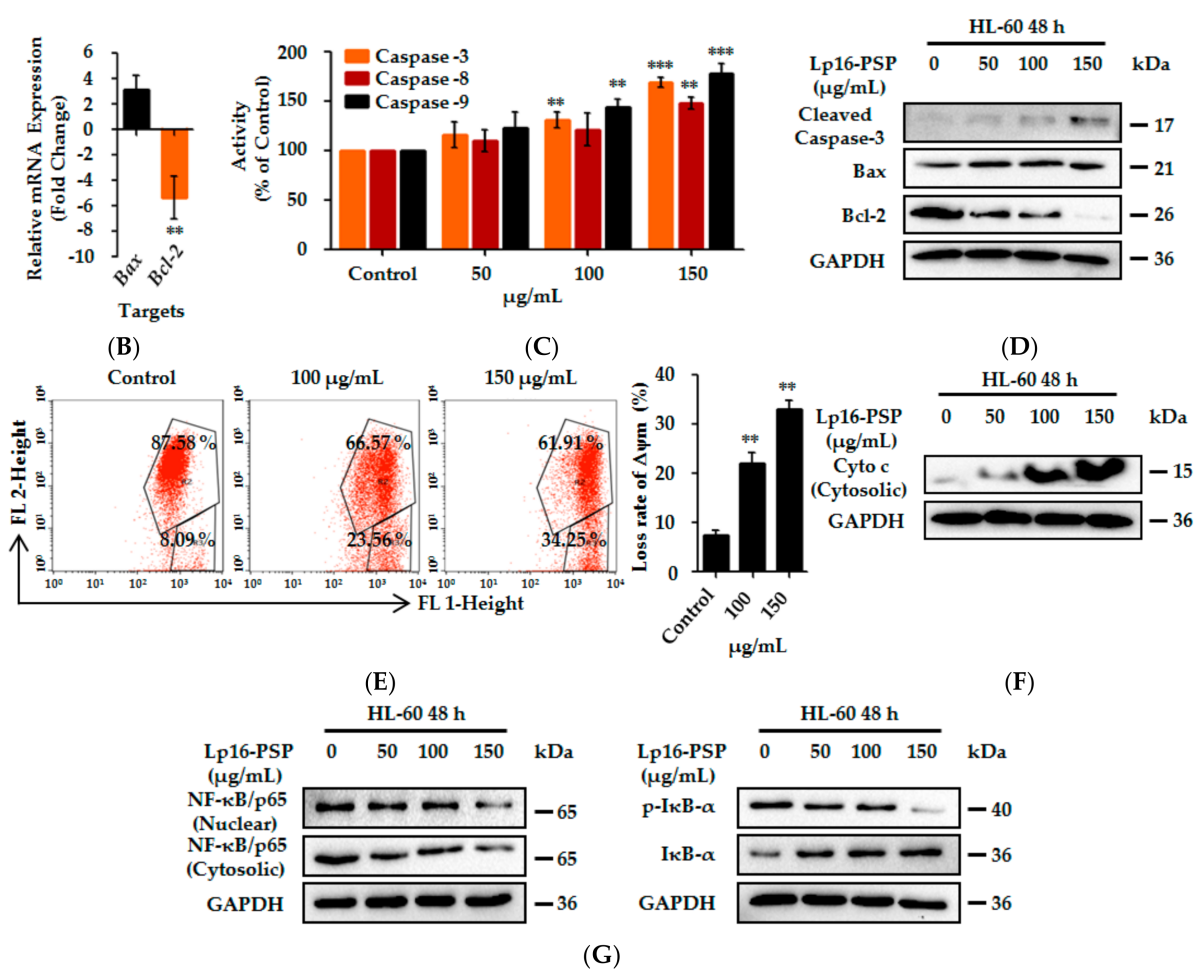
Induction of apoptosis and abrogation of constitutive transcription factor nuclear factor kappa B (NF-κB) activation by Lp16-PSP in acute promyeloid leukemia (HL-60 cells). (**A**) Apoptosis was analyzed by Annexin-V/PI staining after 48 h treatment of HL-60 cells with indicated concentrations of Lp16-PSP. Left, a histogram of the results from one representative experiment treated with 0, 50, 100 and 150 µg/mL of Lp16-PSP after 48 h treatment. The lower left (Annexin V−/PI−) and lower right (Annexin V+/PI−) quadrants are showing the viable cell population and the cells at the early apoptosis respectively, and the upper-right quadrant (Annexin V+/PI+) is showing the cell population at the late apoptosis. Right, a number of cells in early and late apoptotic phase were calculated. The data are expressed as means ± SD (Standard Deviation) (* *p* < 0.05, ** *p* < 0.01) of the three independent experiments; (**B**) The effect of Lp16-PSP on the expression of *Bax*, a pro-apoptotic, and *Bcl-2*, an anti-apoptotic, genes, after 48 h of treatment, as evaluated by qRT-PCR. The mRNAs under investigation from the test were normalized to *GAPDH* (Glyceraldehyde 3-phosphate dehydrogenase) and plotted as the fold change to the mRNA of control untreated cells, defined as 1. The data expressed here are mean ± SD of the three individual experiments (** *p* < 0.01; (**C**) The colorimetric analysis of caspase-3, -8, and -9 after treatment with an indicated concentration of Lp16-PSP for 48 h. The data reported here are the mean ± SD of three independent experiments each performed in triplicate (** *p* < 0.01, *** *p* < 0.001); (**D**) Western blot analysis of the cleavage of caspase-3, Bax, and Bcl-2 after treatment with different concentrations (0, 50, 100 and 150 µg/mL) of Lp16-PSP for 48 h, using GAPDH as an internal control. Western blots are representative of the three independent experiments; (**E**) The loss of mitochondrial membrane potential in HL-60 cells after treatment with indicated concentrations of Lp16-PSP for 48 h. Left, results from one representative experiment of HL-60 cells treated with indicated concentrations of Lp16-PSP. Right, the loss rate of mitochondrial membrane potential as compared with the control. The data reported here are mean ± SD (** *p* < 0.01) of three separate experiments. (**F**) The release of cytochrome c detected by western blotting after treatment with indicated concentrations of Lp16-PSP for 48 h, using GAPDH as an internal control; (**G**) Western blot analysis of the translocation inhibition of NF-κB into the nucleus after treatment with indicated concentrations of Lp16-PSP for 48 h, using GAPDH as an internal control. Western blots are representative of three independent experiments.

Another hallmark of apoptosis is the externalization of phosphatidylserine on the cell membrane prior to the loss of cell membrane integrity [33,37], which can be monitored by annexin V/propidium iodide (AV/PI) staining [32,38]. HL-60 cells after treatment with various concentrations (0, 50, 100 and 150 µg/mL) of Lp16-PSP for 48 h were analyzed for the induction of apoptosis by using annexin V/propidium iodide (AV/PI) staining. After 48 h treatment, the percentage of apoptotic cells (including early and late apoptotic cells) increased with the concentration of Lp16-PSP from 3.51% to 45.61% (Figure 2A). Statistical analysis showed that the percentage of cells in late apoptosis stage was significantly higher than the control group upon Lp16-PSP (100 and 150 µg/mL) treatment for 48 h. These results suggested that the HL-60 cell death by Lp16-PSP is through the induction of apoptosis.

**Figure 3:**
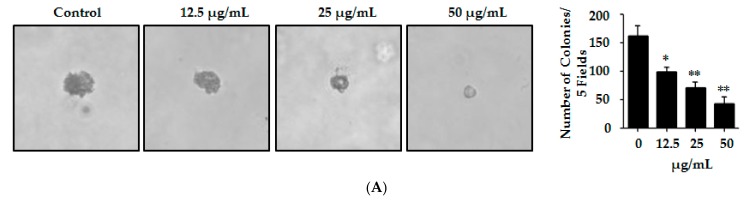

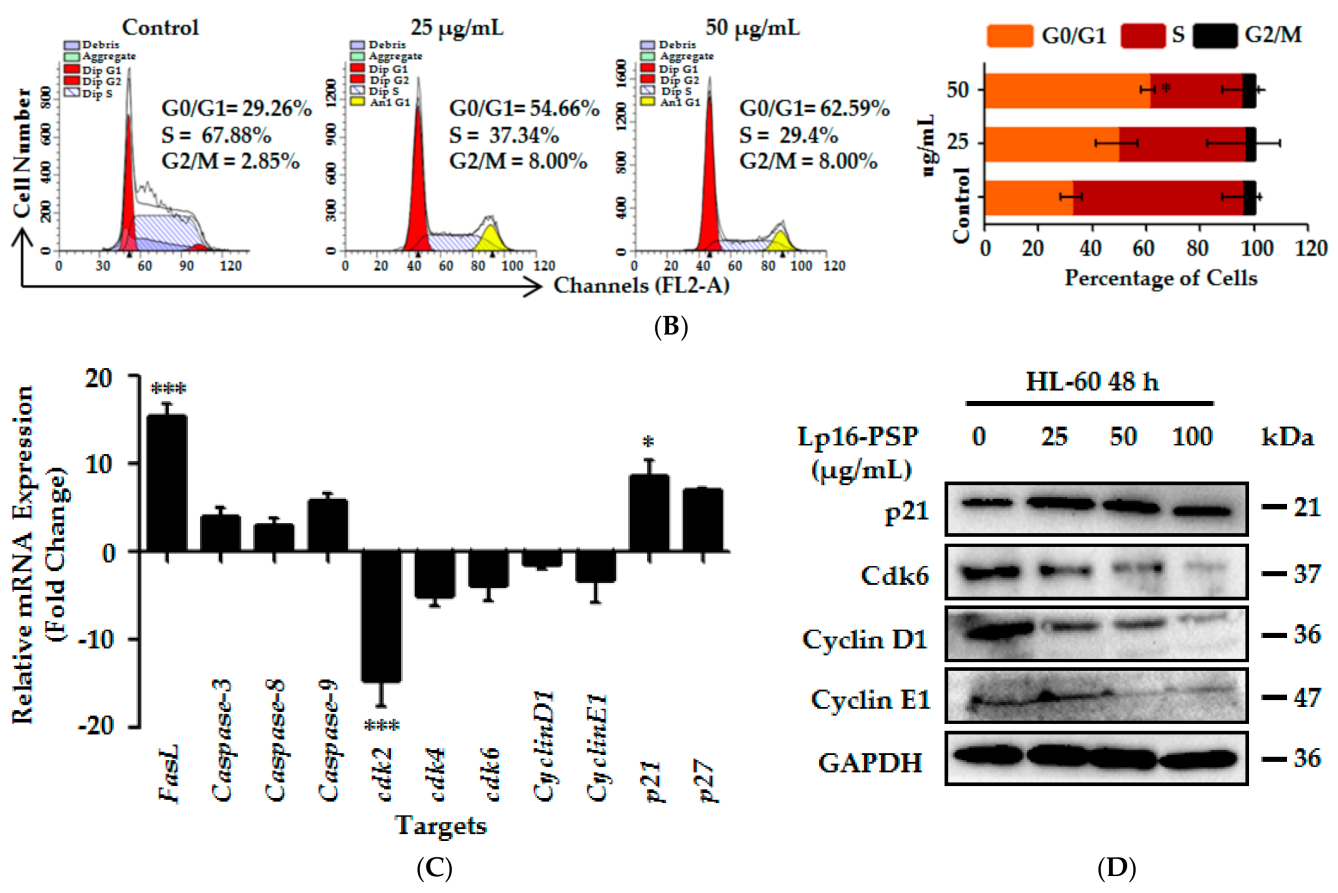
Lp16-PSP suppressed the anchorage-independent growth and Induced p21^WAF1/CIP1^-mediated G_1_ cell cycle arrest in HL-60 cells. (**A**) The effect of non-toxic concentrations of Lp16-PSP (0, 12.5, 25 and 50 µg/mL) on colony formation (anchorage-independent growth) of HL-60 cells was evaluated at 40× magnification and a number of colonies were counted as described in Materials and Methods. The data reported here is the mean ± SD of three separate experiments, * *p* < 0.05, ** *p* < 0.01; (**B**) HL-60 cells untreated and treated with various concentrations of Lp16-PSP for 48 h were analyzed for DNA content using flow cytometry. To the left are the results of the one representative experiment indicating the distribution and percentages of cells in G0/G_1_, S and G2/M phase. To the right is the graphical presentation of the distribution and percentages of cells in different phases of the cell cycle. The data presented here are the means ± SD of three independent experiments where * *p* < 0.05; (**C**) qRT-PCR analysis of apoptosis- and cell cycle-related genes. The data presented here are the means ± SD of two independent experiments each run in triplicate, where * *p* < 0.05, *** *p* < 0.001; and (**D**) Western blot of cell cycle-related proteins after Lp16-PSP treatment with indicated concentrations for 48 h. GAPDH was used as the internal control. Western blots are representative of three independent experiments.

Apoptosis occurs through two main pathways: the Fas death receptor-triggered extrinsic pathway [39] and the mitochondrial-mediated or intrinsic pathway [40]. The initiator caspases, i.e., caspase-8 and -9, upon activation, causes the activation of caspase-3, -6, and -7, which results in the cleavage of the cytoskeleton and nuclear protein, ultimately leading to apoptosis [41]. The Bcl-2 family of proteins also plays a central role in intrinsic apoptosis pathway by binding with Bax and preventing the mitochondrial pore formation and the release of cytochrome c [42]. On the other hand, pro-apoptotic Bax expression causes the induction of apoptosis [43]. Thus, in order to characterize the Lp16-PSP induced apoptosis, we evaluated various apoptosis-related genes, such as *Bax*, *Bcl-2*, *Caspase-3*, *Caspase-8*, *Caspase-9*, and *FasL* after treatment with Lp16-PSP by using qRT-PCR. Treatment with Lp16-PSP (IC_50_ concentration) resulted in the significant up- and downregulation of *FasL* and *Bcl-2* transcripts, respectively, together with increased expression of *Bax*, *Caspase-3*, *-8*, and *-9* (Figure 2B and Figure 3C). Furthermore, the activation of caspase-8 and -9 (initiator caspases) and caspase-3 (effector caspase) was confirmed by the colorimetric assay performed after 48 h of treatment with different concentrations of Lp16-PSP, which showed the activation of caspase-8, -9, and -3 in a dose-dependent fashion (Figure 2C). In addition, Western blot analysis also revealed the cleavage of caspase-3 and the dose-dependent increase and decrease in the expression of Bax and Bcl-2 proteins, respectively (Figure 2D). Increased expression of Bax results in the increased cytochrome c release, that is associated with the mitochondrial damage and intrinsic pathway [44,45]. In our study, Lp16-PSP has resulted in the significant mitochondrial membrane potential loss which ultimately resulted in the release of cytochrome c from mitochondria into the cytosol (Figure 2E,F). All these findings suggest that Lp16-PSP has triggered both extrinsic and intrinsic apoptosis pathways, however, a detailed investigation is required to further characterize the Lp16-PSP induced apoptosis in terms of signal transduction pathway(s) responsible for these outcomes.

### 2.4. Lp16-PSP Inhibits the Consecutive Translocation of NF-κB/p65 into the Nucleus in HL-60 Cells

The transcription factor nuclear factor kappa B (NF-κB) initially identified in the nucleus of B cells, has been shown to be constitutively activated in hematological malignancies, including acute promyeloid leukemia [46,47,48,49,50]. The NF-κB has a role in oncogenesis by having the ability to regulate the expression of a plethora of genes that are involved in the apoptosis, cell survival, proliferation, inflammation, tumor metastasis, and angiogenesis [51]. Therefore, we speculated that the Lp16-PSP mediated its anticancer effect by modulating the activity of NF-κB.

Western blot analysis was performed in order to evaluate the effect of Lp16-PSP on NF-κB/p65 and IκB-α in acute promyeloid leukemia (HL-60 cells). Figure 2G depicts the dose-dependant decrease in the NF-κB/p65 protein levels in both cytosolic and the nuclear fraction upon Lp16-PSP treatment for 48 h. Consistent with these outcomes, a decrease in the protein level of NF-κB/p65 was found to be associated with the concentration-dependant increase and decrease of IκB-α and phospho-IκB-α in the cytosolic fractions respectively (Figure 2G). Taken together, our findings suggest that treatment with Lp16-PSP has resulted in the inhibition of constitutive translocation of NF-κB/p65 into the nucleus in HL-60 cells by decreasing the phospho-IκBα levels.

### 2.5. Lp16-PSP Suppresses the Anchorage-Independent Colony Formation of HL-60 Cells

Metastatic malignant cells have the ability to resist the detachment-induced death, which helps them to grow and survive during the period of their dissemination [52]. HL-60 has been reported previously to have the outstanding property of proliferating and forming sizable colonies in soft agar from a single cell [53]. In order to determine whether Lp16-PSP can affect the HL-60 colony formation, HL-60 cells were mixed with soft agar and cultured until the development of visible colonies. Colonies so formed in the agar were counted carefully. Our results indicated the significant dose-dependent effect of Lp16-PSP on HL-60 colony formation in semisolid agar. These results also indicate that Lp16-PSP treatment resulted in the suppression of HL-60 colony formation without any apparent cytotoxicity at the concentrations of Lp16-PSP used (Figure 3A).

### 2.6. Lp16-PSP Induces G_1_ Phase Cell Cycle Arrest in HL-60 Cells

Lp16-PSP resulted in the suppression of HL-60 cell growth in a dose- and time-dependent fashion [15]. In order to verify that the suppression of growth is due to the disruption of the cell cycle, flow cytometry was performed for the analysis of cell cycle distribution after Lp16-PSP treatment at different concentrations (0, 25 and 50 µg/mL). As shown in Figure 3B, Lp16-PSP treatment at low doses, i.e., 25 or 50 µg/mL, resulted in an increased cell population in G_1_ phase, with approximately 49% and 60% cells in the G_1_ phase, respectively, in comparison to approximately 32% in control after 48 h treatment. This increase G_1_ phase cell population was observed to be related to the decrease in the S phase population, however, upon treatment, the G_2_/M phase remained unchanged. These findings suggest that Lp16-PSP at low doses caused HL-60 growth suppression by modulating the progression of cell cycle without the induction of apoptosis.

### 2.7. Lp16-PSP Induced p21^WAF1/CIP1^ Mediated G_1_ Cell Cycle Arrest in HL-60

Furthermore, we investigated the effect of Lp16-PSP on different cell cycle regulatory genes at the mRNA and protein levels after 48 h of treatment. As p21 is well-recognized as the universal inhibitor of cyclin-cdk complexes [54,55,56] we assessed the expression of p21, and p27, cyclins (cyclin D1, cyclin E1) and cdks (cdk2, cdk4, cdk6) that are operative in the G_1_ phase of the cell cycle by qRT-PCR. Treatment of HL-60 cells with Lp16-PSP (IC_50_ concentration) resulted in the down-regulation of *cdk2*, *cdk4*, *cdk6*, *cyclin D1*, and *cyclin E1*, with the significant upregulation of CDK inhibitory genes (*p21*), however, increased expression of *p27* was also observed (Figure 3C). Moreover, Western blotting revealed the dose-dependent decrease and increase in the expression of cdk6, cyclin D1, cyclin E1 and p21, respectively (Figure 3D). Therefore, these results suggested that proliferation inhibition and G_1_ arrest in HL-60 is mediated by the upregulation of p21 that is involved in the progression of the cell cycle from the G_1_-S phase.

It has been uncovered by the molecular analysis of human tumors that cell cycle controllers are often mutated in the majority of the malignancies; thus, the control of cell cycle progression in cancer is thought to be one of the compelling strategies to battle cancer [57,58]. Our data suggested the potential application of Lp16-PSP in combating cancers with deregulated cell cycle components.

## 3. Discussion

We have reported the cloning, expression, and selective in vitro anticancer activity of Lp16-PSP from *L. edodes* strain C_91-3_ against a panel of human cancer and normal cell lines, and the HL-60 cell line was identified as one of the most sensitive cell lines used [15]. Thus, for further investigations, we have used human acute promyeloid leukemia (HL-60 cells) as the model cancer. In this study, our findings demonstrated that high doses of Lp16-PSP resulted in the induction of morphological changes (Figure 1A), nuclear chromatin condensation (Figure 1B), cleavage of chromosomal DNA in a DNA ladder-like pattern (Figure 1C), accumulation of a significant percentage of apoptotic cells in the lower right (Annexin V+/PI−) and upper right (Annexin V+/PI+) quadrants (Figure 2A), and the loss of mitochondrial membrane potential (Figure 2E) in HL-60 cells.

Initially, the expression of apoptosis- and cell cycle-related genes was evaluated by qRT-PCR. The results showed that Lp16-PSP treatment (IC_50_ concentration) for 48 h resulted in the upregulation of *Bax*, *caspase-3*, *caspase-8*, *caspase-9*, *FasL*, *p21,* and *p27* with the downregulation of *Bcl-2*, *cdk2*, *cdk4*, *cdk6*, *cyclin D1*, and *cyclin E1* transcripts in HL-60 cells (Figure 2B and Figure 3C). Activation of the initiator (caspase-8, caspase-9) and the effector caspases (caspase-3) was confirmed by colorimetric analysis, where Lp16-PSP resulted in the dose-dependent increase in the activity of caspase-3, -8, and -9 (Figure 2C). Increased expression of FasL and activation of caspase-8 clearly demonstrated that the extrinsic pathway might be involved in Lp16-PSP-induced apoptosis. Furthermore, Western blot analysis revealed the cleavage of caspase-3, increased expression of Bax, Bcl-2, and the release of cytochrome c after treatment with Lp16-PSP in a dose-dependent fashion (Figure 2D,F). These findings suggested the involvement of intrinsic pathway in Lp16-PSP induced apoptosis in HL-60 cells. On the other hand, low doses of Lp16-PSP resulted in the anchorage-independent growth inhibition (Figure 3A) and the induction of G_1_ cell cycle arrest as demonstrated by the results of flow cytometry (Figure 3B). In addition, increased expression of the universal inhibitor of cyclin-cdk complexes (p21^WAF1/CIP1^) together with the decreased expression of cyclin D, E, and cdk6 was also confirmed by Western blot analysis (Figure 3D). These findings suggest that Lp16-PSP resulted in the induction of p21^WAF1/CIP1^ mediated G_1_ cell cycle arrest in HL-60 cells.

The transcription factor nuclear factor kappa B (NF-κB) has been reported to have a role in apoptosis, immortalization, angiogenesis, invasion, and metastasis, and have also been shown to be constitutively active in some of the cancer types, including acute myeloid leukemia [51,59,60]. Presently, five mammalian NF-κB family members have been identified and reported including NF-κB1 (p50/p105), NF-κB2 (p52/p100), RelA (p65), RelB and c-Rel [59,61]. In the inactive state, the nuclear localization signal (NLS) of NF-κB is masked by IκB and so the NF-κB complex remains in the cytoplasm. Upon stimulation, the IκBs gets phosphorylated and degraded by ubiquitination, allowing freed NF-κB to translocate to the nucleus and transactivating the κB responsive elements [60,62]. This activation of NF-κB results in the transcription of genes that are involved in antiapoptotic (*Survivin*, *Bcl-xL*, *Bcl-2*, *cIAPs*, *c-FLIP*) proliferative (*Cyclin-D1*, *myc*), proinflammatory (*iNOS*, *COX-2*), invasive (*MMP-9*), and angiogenic (*VGEF*) activities [59,63,64,65]. Highlighting the fact that the agents that prevent the inactivation of NF-κB may serve as a potential arsenal for combating cancer, several lines of evidence have shown that the mushroom-originated compounds exhibits their anticancer effect by modulating different nodes of signal transduction pathways, including NF-κB [66]. Herein, our data indicated that Lp16-PSP from *L. edodes* strain C_91-3_ has resulted in the inhibition of NF-κB translocation into the nucleus by reducing the phospho-IκB-α levels (Figure 2G). As mentioned above, Lp16-PSP resulted in the reduced levels of *Bcl-2* and *cyclin-D1* genes, which are involved in apoptosis and proliferation, respectively, and comes downstream of NF-κB, further highlighting that the anticancer effect of Lp16-PSP is mediated by the inhibition of NF-κB. In spite of all these findings, at this stage it is difficult to conclude the possible molecular mechanism of the action of Lp16-PSP and further support our experimental findings on the basis of previously reported information associated with the other members of YjgF/YER057c/UK114 family. However, antineoplastic, ribonuclease, inhibition of protein synthesis, and antiviral activities of the other members of the YjgF/YER057c/UK114 protein family has been reported [16,19,22,23,24,67] and, afterward, it was proven that translation inhibition was driven by endoribonucleolytic activity. In addition to this, various ribonucleases have been reported to have selective anticancer properties and onconase being one of the well-studied ribonucleases that has been reported to exert its anticancer effect by downregulating and suppressing the NF-κB activity via targeting dsRNA, a cofactor of dsRNA-dependant protein kinase R (PKR), an enzyme that results in the phosphorylation of IκB [68,69,70,71,72,73]. In the recent past, onconase has also been reported to induce the beclin1-mediated autophagic cell death and sensitizing pancreatic cancer to gemcitabine via activating Akt/mTOR pathway in a reactive oxygen species (ROS)-dependent manner [74,75].

Based on previously available information related to YjgF/YER057c/UK114 family members, antitumor ribonucleases, and our findings in this study, we believe that Lp16-PSP might have exerted its in vitro anticancer activity through the induction of reactive oxygen species (ROS) generation, RNA degradation, and/or through the inhibition of protein synthesis. However, this study gives a very preliminary indication of the potential application of Lp16-PSP in combating cancer and the precise mechanism of the Lp16-PSP-mediated NF-κB inhibition in human acute promyeloid leukemia (HL-60 cells) is not clear; thus, further investigations are needed and our future studies will be focused on the shortcomings of this study, including the in vitro enzymatic (endoribonuclease) activity of Lp16-PSP, substrate/target identification (tRNA, rRNA, mRNA, microRNA, or lncRNA), mode of entry into the cell (specific receptors mediated endocytosis), resistance to ribonuclease inhibitors, site of action (nucleus/cytosol), and interaction with intracellular molecules. So that the detailed molecular mechanism in both in vitro and in vivo models can be explored, the activity of Lp16-PSP can be compared with the already-known antitumor ribonucleases and most important therapeutic implications of Lp16-PSP can be made possible in the near future after concrete preclinical and clinical trials.

## 4. Material and Methods

### 4.1. Expression of the Recombinant Protein Lp16-PSP

The Latcripin-16 (designated as Lp16-PSP) is one of the registered proteins of *Lentinula edodes* C_91-3_ from our laboratory, with the accession no. AHB81541. The expression and recovery of the bioactive form of 32 kDa Lp16-PSP protein were accompanied as described previously [15]. Briefly, for routine experimentation, Lp16-PSP was expressed at 37 °C after induction with 0.5 mM IPTG for 4 h, in Rosetta gami (DE3), using pET32a (+) as the expression vector. Solubilization of the protein was achieved by mild solubilization buffer containing 2 M urea by the freeze-thaw method. Purification and refolding were done under optimized conditions. The finalized protein thus obtained after extensive dialysis was concentrated by using PEG 20,000. At each step, Lp16-PSP was qualitatively and quantitatively analyzed by SDS-PAGE (sodium dodecyl (lauryl) sulfate-polyacrylamide gel electrophoresis) and BCA (bicinchoninic acid), respectively, and then used subsequently for biological assays.

### 4.2. Human Leukemia HL-60 Cells and Culture Conditions

Human acute promyeloid leukemia cell line (HL-60) was obtained from the Shanghai cell bank, Chinese Academy of Sciences (Shanghai, China) and was grown in RPMI medium containing 10% fetal bovine serum, penicillin (100 units/mL), and streptomycin (100 μg/mL) at 37 °C in a humidified atmosphere containing 5% CO_2_. HL-60 cells were maintained in exponential growth, and they were passaged when cell confluency reached ~80%.

### 4.3. Antibodies, Kits, and Reagents

Bax, Bcl-2, cleaved caspase-3, cdk6, cytochrome c, cyclin D1, cyclin E1, GAPDH, IκB-α, NF-κB/p65, p21, secondary antibodies, and RIPA buffer were from Proteintech (Wuhan, China). Phospho-IκB-α (Ser 32) primary antibody was purchased from Cell Signaling Technology (Cell Signaling Technology, CST, Beverly, MA, USA). The Annexin-V/PI Kit, mitochondrial membrane potential kit, Hoechst 33258 assay kit, DNA fragmentation kit, and Caspase-3, -8, and -9 kits were purchased from keyGEN BioTECH (Nanjing, China). The nuclear and cytosolic protein extraction kit was purchased from Beyotime (Nanjing, China). The qRT-PCR kit was purchased from Transgene (Shenzhen, China). All other reagents and chemicals were purchased from standard commercial sources.

### 4.4. Phase Contrast Imaging

The HL-60 cells (2 × 10^5^ cells/well) after overnight incubation at 37 °C in 12 well plate was washed with PBS once and grown for 48 h in culture media with previously established doses of Lp16-PSP (0, 50, 100 and 150 µg/mL). After treatment, cell morphology was examined and photographed by using a phase contrast microscope [76].

### 4.5. Hoechst 33258 Staining

HL-60 cells (2 × 10^5^ cells/well) in 12 well plate after treatment with indicated concentrations of Lp16-PSP for 48 h were washed twice with cold buffer A provided with the kit (Hoechst 33258 Detection Kit, Keygen, Nanjing, China) and fixed by using 4% formaldehyde solution at 4 °C for 10 min. Cells were washed again with buffer A and stained with 100 μL of Hoechst 33258 working solution for 10 min at room temperature. After washing with water and air dry, cells were observed with a fluorescence microscope.

### 4.6. DNA Fragmentation Assay

HL-60 cells (5 × 10^6^ cells) were treated with different concentration of Lp16-PSP (0, 50, 100 and 150 µg/mL), and after 48 h of treatment DNA fragmentation assay was done following the manufacturer’s instructions (DNA Fragmentation Assay Kit, KeyGen). Briefly, cells after treatment were collected in 1.5 E.P tubes and washed with PBS. Cells were lysed with the lysis buffer and enzymes provided with the kit, DNA was then precipitated and washed with 70% ethanol. Equal amount of each DNA sample was mixed with the loading buffer and run on 1.5% agarose gel and image were captured by a ChemiDco^TM^ XRS + Imager-Bio-Rad.

### 4.7. Colorimetric Analysis of Caspase-3, -8, and -9

Caspase activities in HL-60 cells after treatment with indicated concentrations of Lp16-PSP for 48 h, were measured by using the commercially available kits (Caspase-3, -8, and -9 Kit, KeyGen). Briefly, cells after treatment with several concentrations of Lp16-PSP for 48 h, were washed twice with PBS and subjected to caspase assay as per manufacturer’s instructions. The activity of the caspase-3, -8, and -9 was normalized and expressed as O.D_Test_/O.D_Control_ × 100.

### 4.8. Apoptosis Analysis Using Annexin-V-FITC/PI Staining

HL-60 cells were treated with different concentrations of Lp16-PSP for 48 h. Thereafter, cells were collected, washed, and stained as per the manufacturer’s instructions (Apoptosis Detection Kit, Keygen). The rate of apoptosis was measured by flow cytometry (FACS-Calibur Cytometer (BD Biosciences, Heidelberg, Germany)) within 1 h.

### 4.9. Mitochondrial Membrane Potential (Δψm) Measurement Using JC-1 Staining by Flow Cytometry

Mitochondrial membrane potential assay was performed as per the manufacturer’s instructions. Briefly, HL-60 cells after treatment with Lp16-PSP (0, 100 and 150 µg/mL) for 48 h were collected after centrifugation and washed twice with PBS. Cells were then incubated with the working solution of JC-1 stain at 37 °C for 30 min. Cells were then collected and resuspended in incubation buffer provided with the kit, and the loss of mitochondrial membrane potential was analyzed by using a FACS-Calibur cytometer (BD Biosciences, Heidelberg, Germany).

### 4.10. Soft-Agar Colony Formation Assay

The evaluation of anchorage-independent growth was done by clonogenicity of cells on soft-agar. Lp16-PSP treated (0, 12.5, 25 and 50 µg/mL) HL-60 cells (1 × 10^3^ cells/well) were mixed with 1.2% agar in growth medium, and plated on top of a solidified layer of 0.3% agar in growth medium, in 6 well plates. Cells were fed every 3 days with growth medium, and colony formation was observed daily under a phase-contrast microscope. A number of colonies were counted in five fields under a microscope at 40× magnification [77].

### 4.11. Cell-Cycle Analysis by Flow Cytometry

HL-60 cells were treated with indicated concentrations of Lp16-PSP for 48 h. After treatment cells were collected, washed with PBS and fixed overnight with 70% ethanol at 4 °C. After fixing, cells were collected by slow centrifugation, washed with ice-cold PBS and resuspended at a concentration of 1 × 10^6^ cells/mL in 5 µg/mL RNase and 50 µg/mL propidium iodide. The cells were then incubated for 30 min at 37 °C and analyzed by using a FACS-Calibur Cytometer (BD Biosciences, Heidelberg, Germany).

### 4.12. Isolation of RNA and Quantitative Real-Time Polymerase Chain Reaction (qRT-PCR)

Expression of the apoptosis- and cell cycle-related genes was determined by quantitative real-time PCR. After treatment of the HL-60 cells with IC_50_ concentration for 48 h, total RNA was extracted from Lp16-PSP treated and control cells with TRIzol reagent (Invitrogen Life Technology Gaithersburg, MD, USA), according to the manufacturers’ instructions. One microgram of RNA was used to generate cDNA by using a Transgene RT reagent kit with gDNA remover. To quantify a number of transcripts, SYBER Green based qPCR was performed with RT master mix (Transgene) using Real-Time PCR System (StepOne^TM^ Applied Biosystems, Singapore). The thermal profile used was as follows: For Reverse transcription 42 °C–15 min, 85 °–5 s, for quantitative PCR 94 °C–30 s, 40× (94 °C–5 s, 60 °C–15 s, 72 °C–10 s). The primer sequences for apoptosis- and cell cycle-related genes are listed in (Table 1). GAPDH was used as an internal control and all the reactions were performed in triplicate. The relative gene expression was calculated by using the 2^−ΔΔ*C*t^ method as described previously [78].

### 4.13. Western Blotting

Lp16-PSP treated and control cells were washed twice with ice-cold PBS and lysed in RIPA (radio immunoprecipitation assay) buffer supplemented with protease inhibitor cocktail (Transgen Biotech, Beijing, China) and phosphatase inhibitor (Transgen Biotech, Beijing, China) on ice for 30 min. Cell lysates were cleared by centrifugation at 14,000 rpm, for 20 min at 4 °C. Preparation of nuclear and cytosolic protein was done by using a nuclear and cytosolic protein extraction kit (Beyotime, Nanjing, China). Protein concentrations were determined by using a BCA kit, and proteins (20–40 µg) were resolved by electrophoresis and transferred to polyvinylidene fluoride (PVDF) membranes (Millipore, Billerica, MA, USA). In order to prevent non-specific antibody binding, membranes were blocked with blocking buffer (5% skimmed milk in TBS-T (20 mM Tris-HCl (pH 7.5), 150 mM NaCl, 0.1% Tween 20)) at room temperature for 1 h. Blots were incubated at 4 °C overnight with the following antibodies diluted with blocking buffer: Bax (catalog no. 23931-1-AP, 1:500), Bcl-2 (catalog no. 12789-1-AP, 1:1000), cleaved caspase-3 (catalog no. 25546-1-AP, 1:500), cyclin D1 (catalog no. 60186-1-AP, 1:2000), cyclin E1 (catalog no. 11554-1-AP, 1:500), cdk6 (catalog no. 14052-1-AP, 1:200), cytochrome c (catalog no. 10993-1-AP, 1:200), GAPDH (catalog no. 23931-1-AP, 1:500), IκB-α (catalog no. 10268-1-AP, 1:500), NF-κB/p65 (Cat # 10745-1-AP, 1:500), Phospho-IκB-α (Cat # 2859, 1:1000), p21 (catalog no. 10355-1-AP, 1:500). After three washes with TBS-T membranes were incubated with the second antibodies: goat anti-rabbit IgG or goat anti-mouse IgG (HRP-conjugated, Proteintech) (catalog no. 23931-1-AP, SA00001-1, 1:500) at room temperature for 1 h. Blots were developed with ECL (Enhanced Chemiluminescence) chemiluminescence detection kit and images were captured by a ChemiDco^TM^ XRS + Imager-Bio-Rad.

### 4.14. Statistical Evaluation

Statistical analysis was done by using GraphPad Prism 5.0 software (La Jolla, CA, USA). All the experiments were performed in triplicate unless otherwise stated. Data were evaluated for significance by using one-way analysis of variance (ANOVA) followed by Tukey’s Multiple Comparison Test.

## 5. Conclusions

In conclusion, our study demonstrated the in vitro anticancer potential of Lp16-PSP a recombinant protein from the mushroom *Lentinula edodes* C_91-3_ using human acute promyeloid leukemia (HL-60 cells) as model cancer. Lp16-PSP exerted its anticancer effect against human promyeloid leukemia (HL-60 cells) by targeting multiple signaling pathways such Fas/FasL-mediated apoptotic pathway, intrinsic apoptotic pathway and by the induction of G1 cell cycle arrest. In addition, Lp16-PSP also resulted in the constitutive translocation inhibition of NF-κB into the nucleus by decreasing the level of phospho-IκBα.

All our findings suggested Lp16-PSP as promising antileukemia agents, however, further investigation on multiple pre-clinical models are needed so that the clinical implication of Lp16-PSP can be made possible in near future.

## Figures and Tables

**Table 1:** qRT-PCR primer sequences.

Genes	Forward 5′ → 3′	Reverse 5′ → 3′
*Bax*	CCCGAGAGGTCTTTTTCCGAG	CCAGCCCATGATGGTTCTGAT
*Bcl-2*	GGTGGGGTCATGTGTGTGG	CGGTTCAGGTACTCAGTCATCC
*Cas3*	AGAGGGGATCGTTGTAGAAGTC	ACAGTCCAGTTCTGTACCACG
*Cas8*	TTTCTGCCTACAGGGTCATGC	TGTCCAACTTTCCTTCTCCCA
*Cas9*	CTCAGACCAGAGATTCGCAAAC	GCATTTCCCCTCAAACTCTCAA
*Cdk2*	GCCATTCTCATCGGGTCCTC	ATTTGCAGCCCAGGAGGATT
*Cdk4*	ATGGCTACCTCTCGATATGAGC	CATTGGGGACTCTCACACTCT
*Cdk6*	CTGCAGGGAAAGAAAAGTGC	CTCCTCGAAGCGAAGTCCTC
*Cyclin D1*	GCTGCGAAGTGGAAACCAT	CCTCCTTCTGCACACATTTGAA
*Cyclin E1*	AAGGAGCGGGACACCATGA	ACGGTCACGTTTGCCTTCC
*FasL*	TGCCTTGGTAGGATTGGGC	GCTGGTAGACTCTCGGAGTTC
*GAPDH*	AATCCCATCACCATCTTCCA	TGGACTCCACGACGTACTCA
*p21*	TGTCCGTCAGAACCCATGC	AAAGTCGAAGTTCCATCGCTC
*p27*	TAATTGGGGCTCCGGCTAACT	TGCAGGTCGCTTCCTTATTCC
